# Triple Lymphoma and Transformation to Diffuse Large B-Cell Lymphoma Finding at the Time of Diagnosis

**DOI:** 10.7759/cureus.43971

**Published:** 2023-08-23

**Authors:** Yaser Rahim, Michael Maroules, Edwin Teehan, Ghassan Bassil, Vanessa Boosahda

**Affiliations:** 1 General Surgery, Aureus University School of Medicine, Passaic, USA; 2 Oncology, St. Mary's General Hospital, Passaic, USA; 3 General and Vascular Surgery, St. Mary's General Hospital, Passaic, USA; 4 Pathology, St. Mary's General Hospital, Passaic, USA

**Keywords:** c-myc oncogene, bcl-6, reed-sternberg cells, lymphoma, hodgkin lymphoma, r-chop chemotherapy, diffuse large b cell lymphoma (dlbcl), lymphoma transformation, primary follicular lymphoma, nodular lymphocyte-predominant hodgkin’s lymphoma

## Abstract

In this report, we present the case of a patient with an uncommon triple diagnosis of (1) follicular (nodular) lymphoma, (2) nodular lymphocyte predominant Hodgkin lymphoma, and (3) diffuse large B-cell lymphoma non-germinal center B-cell (non-GCB) subtype. Although transformation of follicular lymphoma and nodular lymphocyte predominant lymphoma to more aggressive forms such as diffuse large B-cell lymphoma is possible; it generally happens many years after diagnosis. Moreover, there have been reported cases of follicular lymphoma with transformation and nodular lymphocyte predominant Hodgkin lymphoma with transformation at the time of diagnosis, but it is very uncommon to see all three present on initial diagnosis. Our patient presented with a large right axillary mass, which, upon excisional biopsy and subsequent histology, showed the aforementioned lymphomas. The patient did not present with a prodrome of any symptoms except intermittent night sweats. The unique aspect of our case is that transformation and all three lymphomas were seen at the time of diagnosis. The R-CHOP (rituximab, cyclophosphamide, doxorubicin, vincristine, and prednisone) chemotherapy regimen is still the standard method of treatment as it has been shown to be effective in treating follicular lymphoma and nodular lymphocyte predominant Hodgkin lymphoma with and without transformation. However, there is insufficient literature on its efficacy when all three are present concurrently.

## Introduction

Follicular lymphoma (FL) is a B-cell lymphoproliferative neoplasm that originates from the germinal center. The diagnosis and morphological assessment of FL is made via lymph node excision and biopsy. Transformation of FL to large cell lymphomas can be seen in up to 60% of patients [1]. Nodular lymphocyte predominant Hodgkin lymphoma (NLPHL) is a rare subtype of Hodgkin lymphoma (HL) encompassing 3%-5% of HL cases. There have been several reports that show a propensity of NLPHL transformation to diffuse large B-cell lymphoma (DLBCL). Transformation has been seen in up to 30% of cases [2]. Based on its unique histopathological and morphological features, NLPHL has been identified as a distinct subtype in the WHO and the Revised European-American classification of lymphomas [3].

The transformation presented in our case belonged to the postgerminal immunohistochemical variant of DLBCL (non-germinal center B-cell [non-GCB] DLBCL), a molecular analog of the activated B-cell (ABC)-DLBCL subtype, which presents an unfavorable prognosis. This subtype is characterized by the constitutional activation of the B-cell receptor signaling pathway and the transcription factor NF-κB (nuclear factor kappa-light chain enhancer of activated B cells).

This article discusses the case of a triple lymphoma finding. Our patient was found to have all three: FL, NLPHL with transformation to DLBCL concurrently at initial diagnosis. Upon review of the literature, we were unable to find any other case reports discussing such a presentation as such, and this may be the first reported case.

## Case presentation

A 36-year-old, Hispanic male patient presented to the general surgery clinic with a large, painless right axillary mass measuring approximately 3 inches in diameter. He had no history of serious illness, and family history of any cancer was unclear. He does not smoke or use any illicit substances and drinks alcohol socially. He was not taking any prescription medication. He appeared well and was not in any distress. On palpation, the mass was hard and mobile with enlarged surrounding lymph nodes. He did not present with weight loss, fatigue, or fever. The only symptom he had prior to diagnosis was intermittent night sweats. Positron emission tomography combined with computed tomography (PET/CT) revealed an increase in the size of multiple right axillary lymph nodes. The two larger lymph nodes were 5.8 cm and 6 cm in diameter, with several smaller ones located medially and posterior to the pectoralis. There were no enlarged lymph nodes elsewhere.

**Figure 1:**
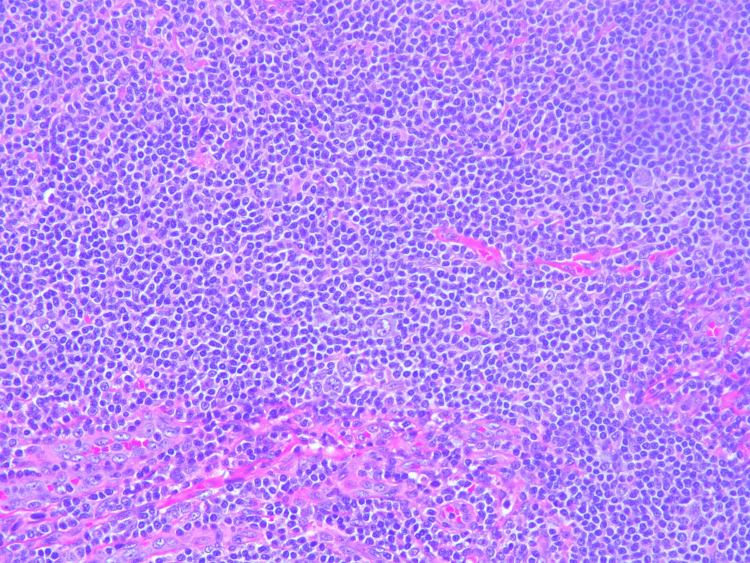
Follicular (nodular) lymphocyte predominant B-cell lymphoma. A high nuclear-cytoplasmic ratio is visible in the tumor cells. Large atypical lymphoid cells are also seen scattered throughout the sample.

**Figure 2:**
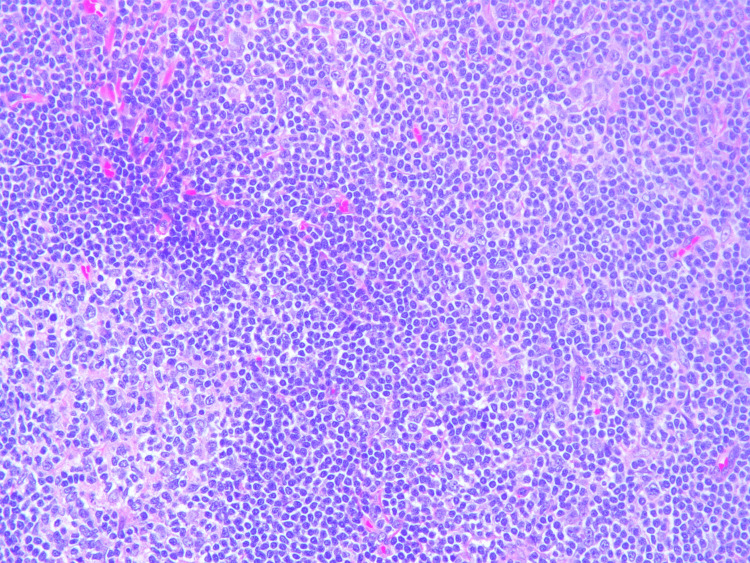
Another sample showing follicular (nodular) lymphocyte predominant B-cell lymphoma with atypical lymphocytes and an area of necrosis.

**Figure 3:**
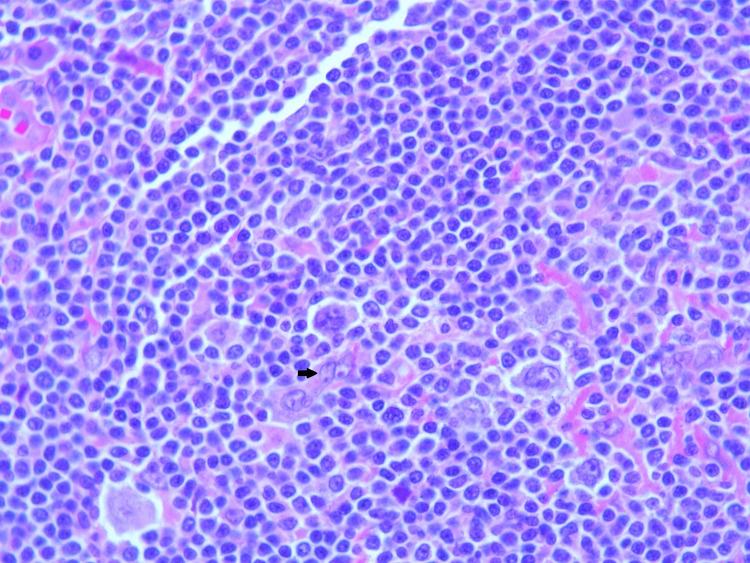
Nodular lymphocyte predominant Hodgkin lymphoma showing characteristic Reed-Sternberg cells (arrow).

**Figure 4:**
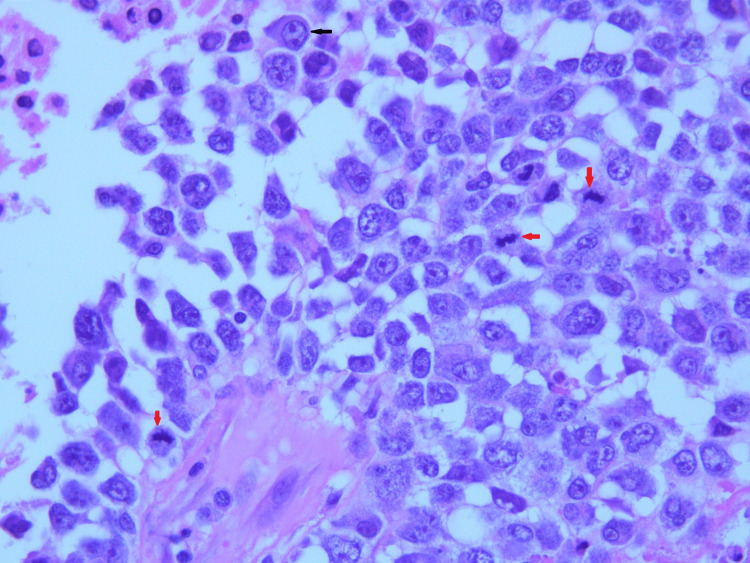
Diffuse large B-cell lymphoma with visible binucleated cell (black arrow) and cells under going mitosis (red arrows).

Lymph node biopsy revealed enlarged lymph nodes with effacement of architecture by a nodular proliferation composed mostly of small lymphocytes and admixed large atypical lymphoid cells consistent with lymphocyte predominant (LP) cells. There were also adjacent nodular areas composed of mostly large atypical lymphoid cells arranged in sheets associated with central necrosis. Histological images directly from our patient's lymph node showing FL can be seen in Figures 1, 2, NLPHL with a characteristic Reed-Sternberg cells can be seen in Figure 3, and transformed DLBCL can be seen in Figure 4 with visible binucleated cells and tumor cells undergoing active mitosis. 

Immunohistochemistry showed that LP cells were positive for CD20, CD45 (variable), PAX5 (variable/weak), OCT2, and Ki-67, and negative for CD15, CD30, ALK1, and BOB1. There were increased numbers of PD1+ T-cells and CD4+ helper T-cells within the nodules and arranged in rosettes surrounding LP cells.

The tumor cells expressed CD45 (LCA), OCT-2, *BCL6*, and expressed increased PD-1, CD4, and variable weak *BCL2*. The tumor cells were negative for CD15 and *BCL1*. FISH diagnosis was positive for extra copies of *MYC*, *BCL2*, *BCL6*, and IgH (nonspecific abnormalities), and was negative for double-hit lymphoma as there was no evidence of translocation or rearrangements. Thus, the diagnosis was established as follicular (nodular) LP B-cell lymphoma (grade 1), NLPHL, (fan pattern A), and focal area of progression to DLBCL (non-GCB subtype).

Four courses of rituximab, cyclophosphamide, doxorubicin, vincristine, and prednisone (R-CHOP) are planned. Serum blood chemistry and LDH will be monitored regularly, and after cycle four, a follow-up PET scan will be administered, likely along with involved field radiation. cycle four, a follow-up PET scan will be administered, likely along with involved field radiation.

## Discussion

The case presented in this article is particularly unique as all three lymphomas (FL, NLPHL, and DLBCL) were present at the initial diagnosis. Another distinguishing factor in this case was the evidence of transformation to DLBCL at the time of diagnosis. Many retrospective studies have been conducted on the incidence of transformation of FL, with limited data from prospective studies. Acker et al. found the risk of FL transformation to be 20% after five years and 60% after eight years of therapy and observation [4]. Ersboll et al. and Montoto et al. defined transformation of FL solely by histology; they reported a transformation rate of 30% and 17% after 5 years and 56% and 28% after 10 years, respectively [4]. Bastion et al. and Al-Tourah et al. defined transformation of FL histologically and clinically; they reported a transformation probability of 22% and 15% at 5 years and 31% and 30% at 10 years, respectively [4]. Another study from March 2004 to March 2007 conducted throughout 265 sites in the US observed 2,652 patients with a confirmed diagnosis of FL. At the time of diagnosis, 47/2,652 (1.8%) patients had transformation to DLBCL [5]. These studies iterate the idea that transformation to more aggressive forms of lymphoma from FL is uncommon and that considerably more uncommon is transformation at the time of diagnosis as seen in our patient.

Kenderian et al. performed an analysis of NLPHL transformation to DLBCL using the Mayo Clinic Lymphoma Database. They identified 222 patients with NLPHL between the years 1970 and 2011 [6]. The cohort consisted of 146 (66%) male patients with a median age at diagnosis of 40 years. The median age at follow-up was 16 years. Transformation to DLBCL occurred within a median time of 35 months, with 17/222 (7.6%) patients presenting with transformation [6]. The rate of transformation was found to be 0.74 per 100 patient-years during the 2,304 patient-years of follow-up. Of the 222 patients, only two patients had concurrent NLPHL and DLBCL (0.90%). The survival rate during five years was 76.4%, and transformation had minimal effect on the overall rate of survival [6].

Extra copies of *BCL6*, *MYC*, and IgH seen in our patient are relatively common in the pathogenesis of lymphoma. The proto-oncogene *BCL6* is most commonly found to be dysregulated and overexpressed as a result of the translocation (3;14) (q27;q32) is seen in 5-10% of DLBCL cases [7]. In addition, somatic mutations leading to disrupted autoregulation of its expression have also been seen in some DLBCL [8]. In our case, extra copies of *BCL6* are seen without any evidence of translocation. The overexpression of the oncogene *BCL2* is also more commonly seen as a result of translocation (14;18) in approximately 20% of DLBCL cases [7]. The increased activity of *BCL2* prevents tumor cells from undergoing apoptosis, thus prolonging their survival. However, the patient presented in our case showed no evidence of *BCL2* translocation or rearrangement, which is an unusual finding. Moreover, overexpression of the *MYC* proto-oncogene was also seen in our patient without the evidence of any translocation or rearrangement, again, making this finding very unusual. The proto-oncogene *MYC* encodes the Myc protein, which is essential for proliferation, metabolism, differentiation, and apoptosis. Overexpression of *MYC* causes increased cell proliferation, angiogenesis, apoptosis, genomic instability, and inhibition of cell differentiation [9]; all of these factors are strongly implicated in the development of several lymphomas including 5-15% of DLBCL cases, as seen in our patient [10].

The presentation of NLPHL is usually localized to the lymph nodes; extranodal, mediastinal, or abdominal involvement is uncommon as evident by the axillary mass present in our case [11]. NLPHL is characterized by a nodular or a nodular and diffuse proliferation of large neoplastic lymphocyte-predominant cells, which are sometimes referred to as lymphocytic and histiocytic or Reed-Sternberg cell variants [12]. In contrast to the Reed-Sternberg cells of classical HL, which express CD15 and CD30, LP cells of NLPHL consistently express the B-cell marker CD20. In addition, the LP cells of NLPHL can also express CD19, CD21 CD45, CD57, CD79 OCT2, *BCL6*, PAX5, PD-1, Ki-67, and MUM1 [11-15].

FL is a B-cell lymphoproliferative neoplasm that originates from the germinal center. The diagnosis and morphological assessment of FL is made via lymph node excision and biopsy. There are two morphologies visible on biopsy; FL displays two types of closely packed follicles: (1) small follicles containing small cleaved cells with nucleoli of variable sizes, referred to as centrocytes, and (2) larger noncleaved cells with moderate cytoplasm, open chromatin, and multiple nucleoli, referred to as centroblasts which are rapidly dividing B cells [16]. Its grading is based on histology and is classified as grade 1-3 (low grade to high grade). The WHO has differentiated the grades based on number of centroblasts, as given in Table 1. Most cases of FL show a (14;18) (q32;q21) translocation resulting in an overexpression of *BCL2*, an antiapoptotic protein. In some cases, dysregulating mutations of *BCL6* are also seen also resulting in an overexpression of *BCL6*, which is required for germinal center formation as seen in our patient [16].

**Table 1:** WHO Classification of FL grades based on the number of centroblasts present. *Grade 3A presents with centrocytes, whereas grade 3B presents with solid sheets of centroblasts.

WHO Classification	Number of Centroblasts
Grade 1	0–5/HPF
Grade 2	6–15/HPF
Grade 3*	>15/HPF

## Conclusions

As evident by the pathology and research presented in this report, it is uncommon to see FL and NLPHL with transformation to DLBCL in a patient at the time of diagnosis. The standard method of treatment is still rituximab, cyclophosphamide, doxorubicin hydrochloride (hydroxydaunomycin), vincristine sulfate (Oncovin), and prednisone (R-CHOP). This combination of therapy still provides the most favorable outcome for patients presenting with singular or multiple lymphomas with or without transformation. Early transformation to DLBCL is a poor prognostic factor, and many patients may still respond well to the R-CHOP chemotherapy regimen. The etiology of genetic mutations involved in the development of lymphoma has been under careful research for many decades and continues to this day. Technological advancements and discovery of new genetic mutations give some hope for the development of more targeted therapies.
